# Unclassified four-repeat tauopathy associated with familial parkinsonism and progressive respiratory failure

**DOI:** 10.1186/s40478-020-01025-1

**Published:** 2020-08-27

**Authors:** Masayoshi Nakano, Yuichi Riku, Kenya Nishioka, Masato Hasegawa, Yukihiko Washimi, Yutaka Arahata, Akinori Takeda, Kentaro Horibe, Akiko Yamaoka, Keisuke Suzuki, Masashi Tsujimoto, Yuanzhe Li, Hiroyo Yoshino, Nobutaka Hattori, Akio Akagi, Hiroaki Miyahara, Yasushi Iwasaki, Mari Yoshida

**Affiliations:** 1 Department of Neurology, National Center for Geriatrics and Gerontology, 7-430 Morioka, Obu, Aichi 474-8522 Japan; 2 Institute for Medical Science of Aging, Aichi Medical University, 1-1 Yazakokarimata, Nagakute, Aichi 480-1195 Japan; 3 Department of Neurology, Juntendo University School of Medicine, 2‑1‑1 Hongo, Bunkyo, Tokyo 113‑8421 Japan; 4 Department of Brain and Neuroscience, Tokyo Metropolitan Institute of Medical Science, 2-1-6 Kamikitazawa, Setagaya, Tokyo 156-8506 Japan; 5 Research Institute for Diseases of Old Age, Graduate School of Medicine, Juntendo University, 2‑1‑1 Hongo, Bunkyo, Tokyo 113‑8421 Japan; 6 Department of Neurology, Nagoya University, 65 Tsurumai-cho, Nagoya, Aichi 466-8560 Japan

**Keywords:** Four-repeat tau aggregation, Familial parkinsonism, Respiratory failure, Autopsy, Postmortem study

## Abstract

We describe an autopsied patient with familial parkinsonism and unclassified four repeat-tau (4R-tau) aggregation. She presented with bradykinesia, truncal dystonia, and mild amnesia at the age of 61 and then exhibited body weight loss (15 kg over 8 months), sleep disturbances, and progressive respiratory failure with CO_2_ narcosis. She died of respiratory failure at the age of 62, 14 months after disease onset. Her brother also showed parkinsonism at the age of 58 and suddenly died 6 months later. Postmortem examination revealed 4R-tau aggregation, which was characterized by neuronal globose-type tangles or pretangles, bush-like or miscellaneous astrocytic inclusions, and coiled bodies. The temporal tip, the striatum, the substantia nigra, the tegmentum of the midbrain, the medullary reticular formation, and the spinal cord were severely involved with tau aggregation. Argyrophilic grains and ballooned neurons were also found in the medial temporal structures, however, extensions of the 4R-aggregations in the case were clearly broader than those of the argyrophilic grains. Western blot analysis of sarkosyl-insoluble fractions from brain lysates revealed prominent bands of tau at both 33 kDa and 37 kDa. Genetic examinations did not reveal any known pathogenic mutations in *MAPT, DCTN*-*1, PSEN*-*1,* or familial or young-onset parkinsonism-related genes. The clinical manifestations, pathologic findings, and biochemical properties of aggregated tau in our patient cannot be explained by argyrophilic grain disease or other known 4R-tauopathies alone. Our results further extend the clinical and neuropathologic spectra of 4R-tauopathy.

## Introduction

Neuronal and glial aggregation of tau protein is known to cause neurodegenerative diseases. Tau protein is classified into three-repeat tau (3R-tau) and four-repeat tau (4R-tau), which are determined by alternative mRNA splicing of exon 10 [1, 10]. Aggregations of 4R-tau are pathologic hallmarks of corticobasal degeneration (CBD), progressive supranuclear palsy (PSP), argyrophilic grain dementia (AGD), globular glial tauopathy (GGT), aging-related tau astrogliopathy (ARTAG), and *microtubule*-*associated protein tau* (*MAPT*) gene mutation-associated familial frontotemporal dementia (FTD) and parkinsonism, whereas neuronal inclusions of 3R-tau are characteristic of Pick’s disease (PiD) [17, 19, 21].

In this report, we describe a patient who presented with progressive parkinsonism, body weight loss, and respiratory failure. Her sibling showed similar clinical manifestations. The proband died 14 months after disease onset, and a postmortem study revealed severe 4R-tau aggregation, which could not be classified into known disease entities.

## Case presentation

### Medical history

**Fig. 1:**
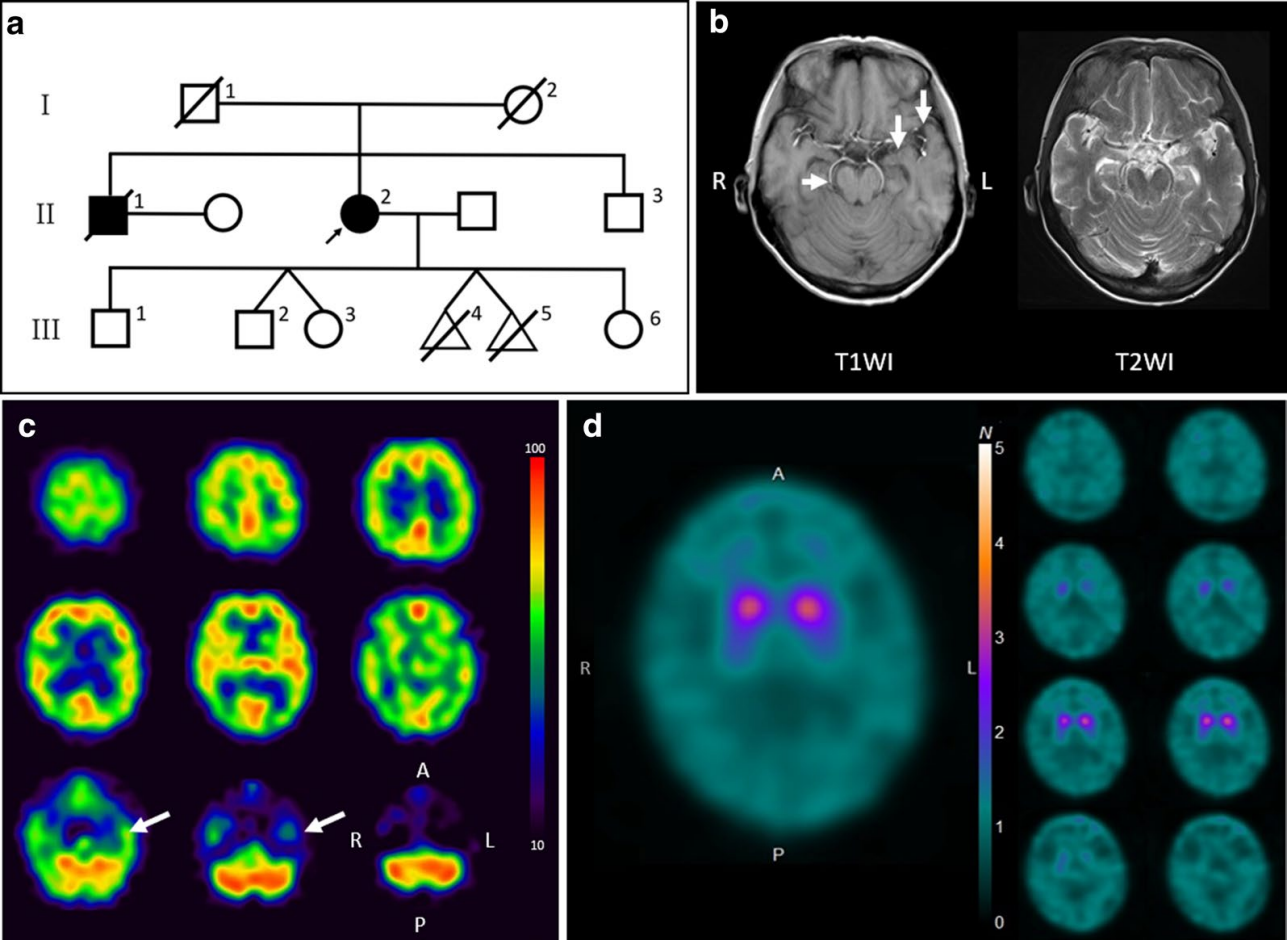
Clinical overview of the patients who presented with parkinsonism and progressive respiratory failure. **a** Our patient is shown as the proband (II-2) with an arrow. Patients with parkinsonism are shown as a filled circle or square for the proband and her elder brother (II-1), respectively. Her fourth (III-4) and fifth (III-5) children were conjoined twins and aborted before being delivered. Oblique lines indicate death. **b** T1- and T2-weighted brain magnetic resonance images (Siemens Magnetom Skyra 3.0 T) show mild atrophy in the midbrain, hippocampus, and temporal lobes, where marked with arrows. **c** I^123^-IMP brain single photon emission computed tomography indicates decreased blood flow in the anterior and medial regions of the left temporal lobes as marked with arrows. **d** The radioactivity in the bilateral putamen of dopamine transporter scintigraphy is depleted and is depicted as dot-like shapes (the specific binding ratio values using the Tossici-Bolt method were 1.72 for the right striatum and 1.62 for the left striatum). Abbreviations: T1WI, T1-weighted brain magnetic resonance images; T2WI, T2-weighted brain magnetic resonance images; A, anterior; P, posterior; R, right; L, left

The proband was a Japanese female (shown as II-2 in Fig. 1a). She had been diagnosed with depression at the age of 50 and recovered at the age of 52 without any medications. At the age of 61, she presented with gait disturbance, dropped head, and amnesia and visited the hospital 6 months after disease onset. Our neurological examinations revealed supranuclear gaze palsy upward, rigidity in the truncus and limbs, clumsiness from diadochokinesis, anteropulsion, masked face, and small voice speech. Limb ataxia and tremor were not observed. Hyperreflexia was observed in the bilateral upper limbs. The Mini-Mental State Examination score was 24 of 30; six points were subtracted from the score in the calculation and recall sections. The indices related to cognitive decline showed 6 of 70 on the Alzheimer’s disease assessment scale-cognitive subscale, 32 of 36 on Raven’s colored progressive matrices test, and 16 of 18 on the frontal assessment battery. All scores were within normal ranges. Brain magnetic resonance images revealed mild atrophies in the midbrain, hippocampus, and anterior portion of the temporal lobe (Fig. 1b). Brain single photon emission computed tomography indicated decreased blood flow in the anterior and medial regions of the left temporal lobe (Fig. 1c). The radioactivity of the bilateral putamen on dopamine transporter scintigraphy (Fig. 1d) was depleted. Meta-iodobenzylguanidine myocardial scintigraphy showed slight depletion of intake (the heart to mediastinum ratios, of which the cutoff value was 2.0, were 1.86 and 1.85 at the early and late registrations, respectively). We made a diagnosis of Parkinsonian syndrome and administered levodopa/benserazide and increased the dose up to 450 mg/day. There was no obvious improvement, and she gradually became less active. Fourteen months after disease onset, she was brought to our hospital. She presented with 15 kg of weight loss since the first visit, drowsiness, cyanosis of the limbs, and myoclonus of the upper limbs. Blood examination revealed secondary polycythemia. Arterial blood gas analysis showed hypercapnia and respiratory acidosis (pH 7.33, pO_2_ 28.8 mmHg, pCO_2_ 90.9 mmHg, base excess extracellular fluid 20.8 mmol/l, HCO_3_^−^ 46.7 mmol/l). She was admitted to the hospital and died of respiratory failure at the age of 62 on the fourth day of admission.

Her elder brother, who is shown as II-1 in Fig. 1a, also presented with parkinsonism, including gait disturbance, masked face, and bradykinesia, at the age of 58. He refused to undergo any diagnostic imaging and was suspected to have Parkinson’s disease according to his symptoms. He died at home 6 months after he first visited our hospital. Her second child, who is shown as III-2 in Fig. 1a, has suffered from depression for 6 years. There was no consanguinity in the family.

### Protocols for neuropathologic, biochemical and genetic analyses

Written informed consent for a postmortem study and genetic analysis was obtained from the patient’s family in compliance with the ethical committee for medical research of Aichi Medical University and Juntendo University, Japan.

The left hemisphere was fixed in 20% formaldehyde for 2 months, embedded in paraffin, and then sectioned into 5 μm thick sections. Hematoxylin and eosin staining, Klüver-Barrera staining, and Gallyas-Braak staining were performed. Primary antibodies for immunohistochemistry were as follows: anti-hyperphosphorylated tau (AT8, mouse monoclonal, 1:3000, Innogenetics, Ghent, Belgium), anti-3R-tau (RD3 8E6/C11, mouse monoclonal, 1:500, Upstate, Lake Placid, NY), anti-4R-tau (RD4 1E1/A6, mouse monoclonal, 1:500, Upstate), anti-phosphorylated TAR DNA binding protein 43 kDa (pTDP-43 s409/410, polyclonal rabbit, 1:5000, Cosmobio, Tokyo, Japan), anti-tyrosine hydroxylase (TH, monoclonal mouse, 1:100, Millipore, Billerica, MA), anti-tryptophan hydroxylase (TrOH, monoclonal mouse, 1:100, Millipore), anti-choline acetyltransferase (ChAT, polyclonal goat, 1:100, Millipore), anti-poly GA (5E9, monoclonal mouse, 1:100, Millipore), and anti-p62 (3/p62 LCK ligand, monoclonal mouse, 1:100, BD Biosciences, Franklin Lakes, NJ) antibodies. Secondary immunolabeling was performed using an EnVision kit (Dako, Glostrup, Denmark). Alexa Fluor 488 and 561 (Thermo Fisher, Waltham, MA) were used for immunofluorescence with the standard protocol.

Tissue lysates of the frozen right prefrontal cortex and caudate nucleus were subjected to western blotting using anti-AT8 and anti-T46 (Thermo Fisher) antibodies, as we described previously [2, 30]. Brain lysates taken from patients with CBD (a male patient who died at the age of 73) and PSP (a male patient who died at the age of 63) were also assessed for comparison. Tau fibrils obtained from sarkosyl-insoluble fractions were analyzed with immune electron microscopy using anti-AT8 antibody and anti-tau C antibody that we generated to label the C-terminal fragment of tau protein.

Gene sequencing analyses were performed at the department of neurology, Juntendo University. We extracted DNA from peripheral blood with a standard protocol. Next, we screened the genes related to Perry syndrome, frontotemporal dementia (FTD), such as *DCTN1*, and *MAPT* by Sanger sequencing and *C9orf72* by the repeat-primed PCR. The technological details and the protocols for *MAPT*, *DCTN*-*1*, and *C9orf72* were described in previous reports [6, 13, 27]. Other genes related to familial or Parkinson’s disease, parkinsonism, and Alzheimer’s disease, including *SNCA*, *PRKN*, *UCHL1*, *PINK1*, *DJ*-*1*, *LRRK2*, *ATP13A2*, *GIGYF2*, *HTRA2*, *PLA2G6*, *FBXO7*, *VPS35*, *EIF4G1*, *DNAJC6*, *SYNJ1*, *DNAJC13*, *CHCHD2*, *GCH1*, *NR4A2*, *VPS13C*, *RAB7L1*, *BST1*, *C19orf12*, *RAB39B*, *PSEN1*, *GRN*, *APP* and *APOE*, were screened by the targeted gene panel sequence with an Ion Torrent System (Thermo Fisher Scientific, Waltham, MA, US). The details of methods are reported previously [11]. The detected variants were confirmed by Sanger sequencing. The panel for sequencing was designed in Ion AmpliSeq Designer [33]. Library preparation was performed using an Ion AmpliSeq Kit for Chef DL8 and an Ion Chef System. Emulsion polymerase chain reaction was performed using an Ion 530 Kit-Chef. The sequences were obtained on an Ion S5 Plus Sequencer using an Ion 530 Chip. Sequence alignment was performed using the Torrent Mapping Alignment Program implemented in v5.10 of Torrent Suite software (Thermo Fisher Scientific).

### Neuropathologic findings

**Fig. 2:**
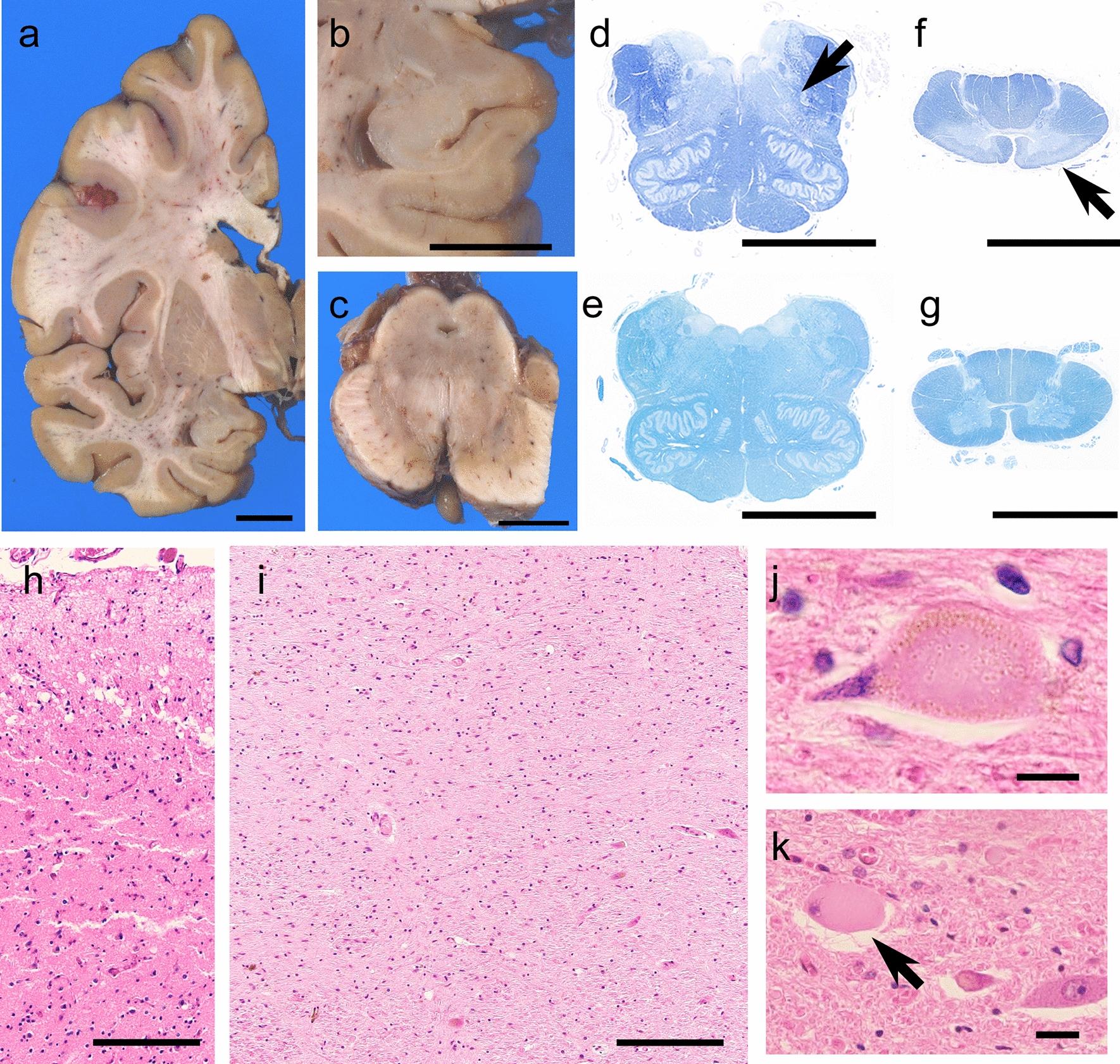
Neuropathologic findings of patient II-2. On a coronal section of the left hemisphere, the basal ganglia and the frontotemporal cortex are not atrophied (**a**), whereas the subiculum shows mild atrophy (**b**). Severe discoloration is observed in the substantia nigra (**c**). The medullary reticular formation is atrophied (**d**, arrow). The fasciculus anterolateralis of the spinal cord shows tract degeneration, whereas the lateral column is spared (e, arrow). For comparison, photomicroscopies from a healthy control (60 years old, female) are also shown (**f**, **g**). The superficial layers of the temporal tip (**h**) demonstrate loss of neurons, astrogliosis, and spongiotic changes. The melanin-containing neurons are severely depleted in the pars reticulate of the substantia nigra (**i**). Ballooned neurons in the temporal cortices (**j**) and the anterior horn of spinal cord (**k**, arrow) are shown. **d**–**g** Klüver–Barrera staining and **h**–**k** hematoxylin–eosin staining were used. Scale bars: **a**–**g** 1 cm, **h**, **i** 100 μm, and **j**, **k** 20 μm

**Fig. 3:**
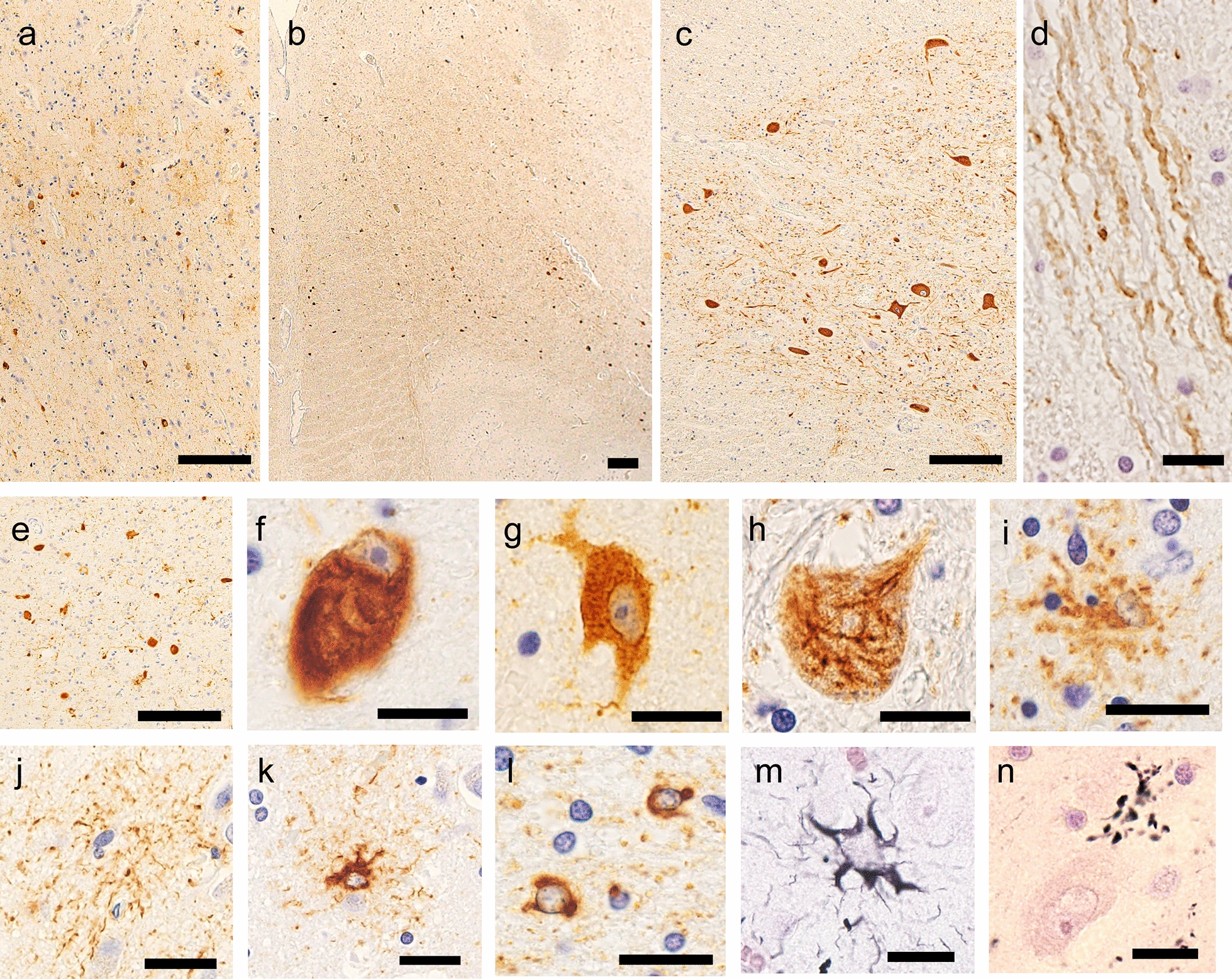
Tau-immunopositive inclusions. Anti-AT8 immunohistochemistry reveals prominent neuronal and astrocytic aggregations in the deep layer of the cerebral cortex (**a**). Tau aggregation is also severe in the medullary reticular formation (**b**) and the anterior horn of the spinal cord (**c**). The intramedullary tract of the hypoglossal nerve shows intraaxonal tau aggregation (**d**). The tau aggregations were four-repeat tau-immunopositive (**a**). The neuronal inclusions comprised globose-type neurofibrillary tangles (**f**) and pretangles (**g**), and a subset of the motor neurons in the spinal anterior horn show tau inclusions with fine, fibrous configurations (**h**). The astrocytes demonstrate miscellaneous aggregations, including bush-like inclusions (**i**), and depositions within the distal (**j**) and proximal foot processes (**k**). Oligodendroglial inclusions are abundant in the cerebral white matter (**l**). Astrocytic inclusions are fundamentally argyrophilic (**m**). The hippocampal pyramidal neurons show argyrophilic grains (**n**). **a**–**d**, **f**–**l** Anti-AT8 immunohistochemistry, **e** anti-RD4 immunohistochemistry, and **m**, **n** Gallyas–Braak staining were used. Scale bars: **a**–**d** 100 μm, **e**, **j**, **k** 20 μm **f**–**i**, **l**–**n** 10 μm

The brain weighed 1260 g before a fixation. The temporal tip and the hippocampus were mildly atrophied. The tegmentum of the brainstem, the medullary reticular formation, and the fasciculus anterolateralis of the spinal cord showed marked atrophy. The substantia nigra showed severe discoloration, whereas the locus coeruleus was retained. The cerebral neocortices, basal ganglia, and cerebellum were not atrophied (Fig. 2a–g). Microscopically, neuronal loss and astrogliosis were prominent in the temporal tip, amygdala, ambient gyrus, and substantia nigra (Fig. 2h–i). Occasional ballooned neurons were observed in those regions and in the anterior horn of the spinal cord (Fig. 2j, k). Immunohistochemistry revealed AT8- and RD4-immunopositive neuronal and glial inclusions to be abundant in the temporal tip, amygdala, CA1, subiculum, ambient gyrus, substantia nigra, tegmentum of the midbrain, reticular formation of the medulla oblongata, hypoglossal nerve nucleus, and anterior horn of the spinal cord (Fig. 3a–e). The neuronal inclusions were globose-type tangles or pretangles (Fig. 3f–g), and a subset of neuronal inclusions in the spinal anterior horn showed fine, fibrous configurations (Fig. 3h). The astrocytes demonstrated miscellaneous aggregations of hyperphosphorylated tau; bush-like granular or thorn-shaped dense aggregations within the proximal or distal portions of the foot processes were observed (Fig. 3i–k). Coiled bodies, which represented oligodendrocytic aggregations of hyperphosphorylated tau, were observed in the white matter (Fig. 3l). These glial 4R-tau aggregations were fundamentally argyrophilic (Fig. 3m). RD3 immunostaining was negative in most regions. Typical tufted astrocytes, astrocytic plaques, or globular glial inclusions were not observed. Argyrophilic grains were observed in the entorhinal cortex, the ambient gyrus, the subiculum, and the CA1, corresponding to stage III of Saito’s grading (Fig. 3n) [29]. Anti-phosphorylated TDP-43 immunohistochemistry revealed occasional dot-like aggregations within the neuronal cytoplasm in the hippocampus and the gray matter of the brainstem. Mislocalization of TDP-43 from the nuclei was not observed. Mild deposition of beta-amyloid was found in the frontal cortex, corresponding to Thal’s phase 1 [31] and grade A of the Consortium to Establish a Registry for Alzheimer’s Disease [24]. Anti-alpha-synuclein immunohistochemistry was negative in the brain, spinal cord, and pericardial sympathetic nerves. The hippocampal granular cells did not show immunostaining of poly-GA dipeptide, although several aggregates of 4R-tau were immunopositive for p62. Group atrophies of the muscle fibers were not observed in the intercostal, biceps, and iliopsoas muscles, the diaphragm, or the tongue. These muscle fibers did not show myogenic atrophies, internal nuclei, or ring fibers. The systemic organs showed no remarkable lesions causative of respiratory failure and death.

**Fig. 4:**
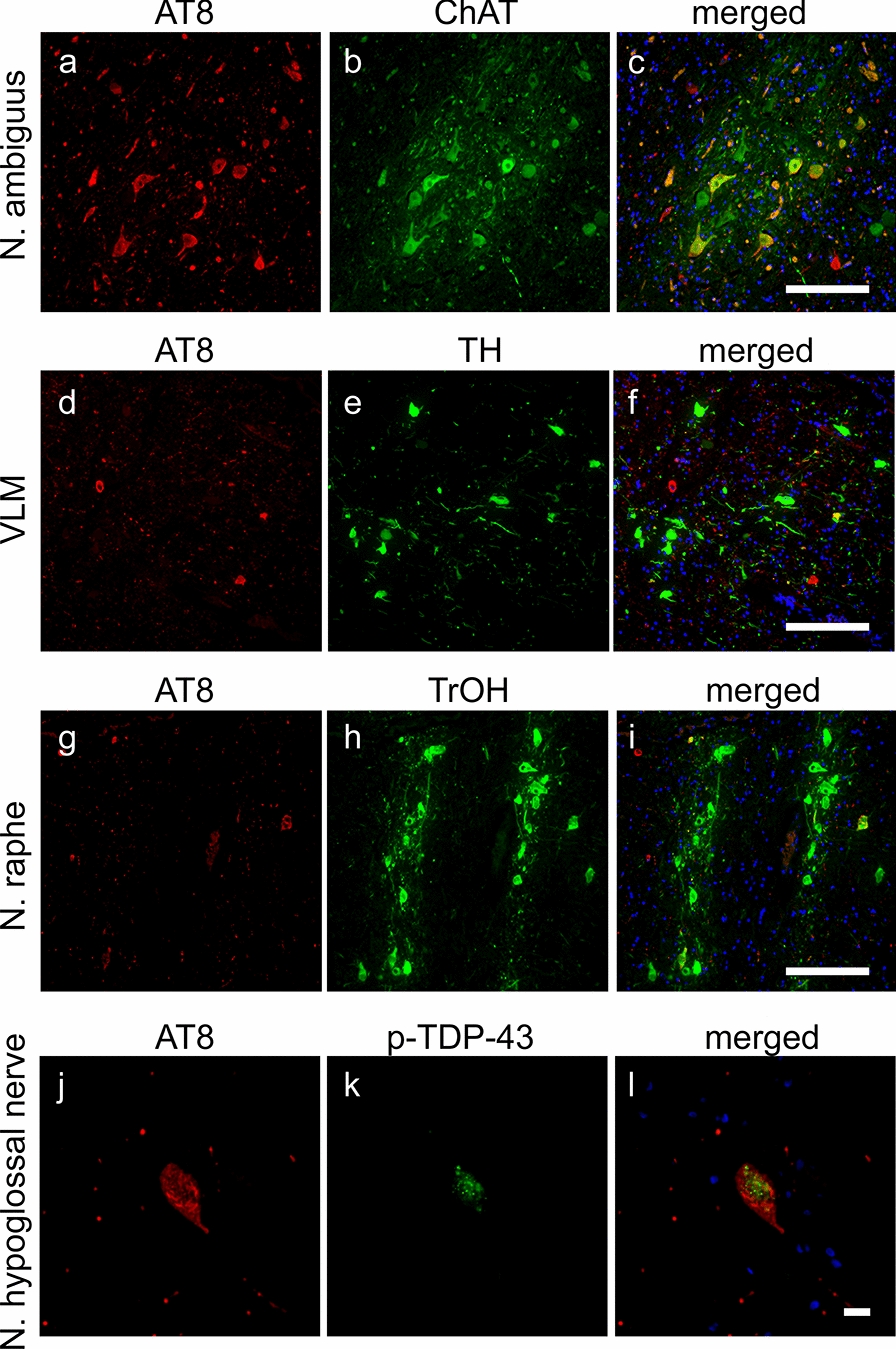
Double immunofluorescence. Panels **a**–**i** show anti-AT8 immunohistochemistry in the medullary reticular formation. Tau aggregations are more prominent in the cholinergic neurons of the nucleus ambiguus (**a**–**c**) than in TH-immunopositive cathecolaminergic neurons in the ventrolateral medullary nucleus (**d**–**f**) or TrOH-immunopositive serotonergic neurons in the raphe nucleus (**g**–**i**). Panels **j**–**l** demonstrate occasional immunopositivity of phosphorylated TDP-43 within neurons containing AT8-immunopositive inclusions of the hypoglossal nerve. Abbreviations: ChAT, choline acetyltransferase; N. ambiguus, nucleus ambiguus; N. hypoglossal nerve, nucleus of the hypoglossal nerve; N. raphe, raphe nucleus; TH, tyrosine hydroxylase; and TrOH, tryptophan hydroxylase. Scale bars: **a**–**c** 50 μm, **d**–**i** 100 μm, and **j**–l 10 μm

Double immunofluorescence for AT8 combined with TH, TrOH, or ChAT was performed to assess tau aggregation within the neurons of the ventrolateral medullary (VLM) nucleus, raphe nucleus, or nucleus ambiguus, respectively, of the medullary reticular formation [28]. The fluorescent signal of AT-8 was more prominently colocalized with ChAT in the nucleus ambiguus than with TH in the VLM nucleus or TrOH in the raphe nucleus (Fig. 4a–i). AT-8-immunopositive neurons occasionally contained dot-like aggregations of phosphorylated TDP-43 (Fig. 4j–l). The distributions of neuronal and glial tau aggregations are summarized in Table 1.

**Table 1:** Distributions of neuronal and glial tau aggregations

Regions	NI	Grain	Glial inclusions
Frontal cortex	+	−	+
Primary motor cortex	++	−	++
Temporal cortex	+	+*	+
Insula	++	−	++
Subiculum/CA1	++	++	++
CA2-4	+	++	+
Dentate gyrus	+	−	+
Parahippocampal cortex	++	++	++
Amygdala	++	+	++
Temporal tip	++	−	++
Parietal cortex	+	−	+
Occipital cortex	+	−	+
Neostriatum	++	−	++
Globus pallidus	++	−	++
Basal nucleus of Meynert	++	−	++
Cerebral white matter	−	−	++
Pars reticulata of substantia nigra	++	−	++
Pars compacta of substantia nigra	+	−	+
Tegmentum of midbrain	++	−	++
Locus ceruleus	+	−	+
Pontine nucleus	+	−	+
Medullary reticular formation	++	−	++
Anterior horn of spinal cord	++	−	++
Dorsal horn of spinal cord	+	−	+

−, negative; +, mild; ++, severe; NI, neuronal inclusions; *, only in the occipitotemporal gyrus

### Outcomes of western blot

**Fig. 5:**
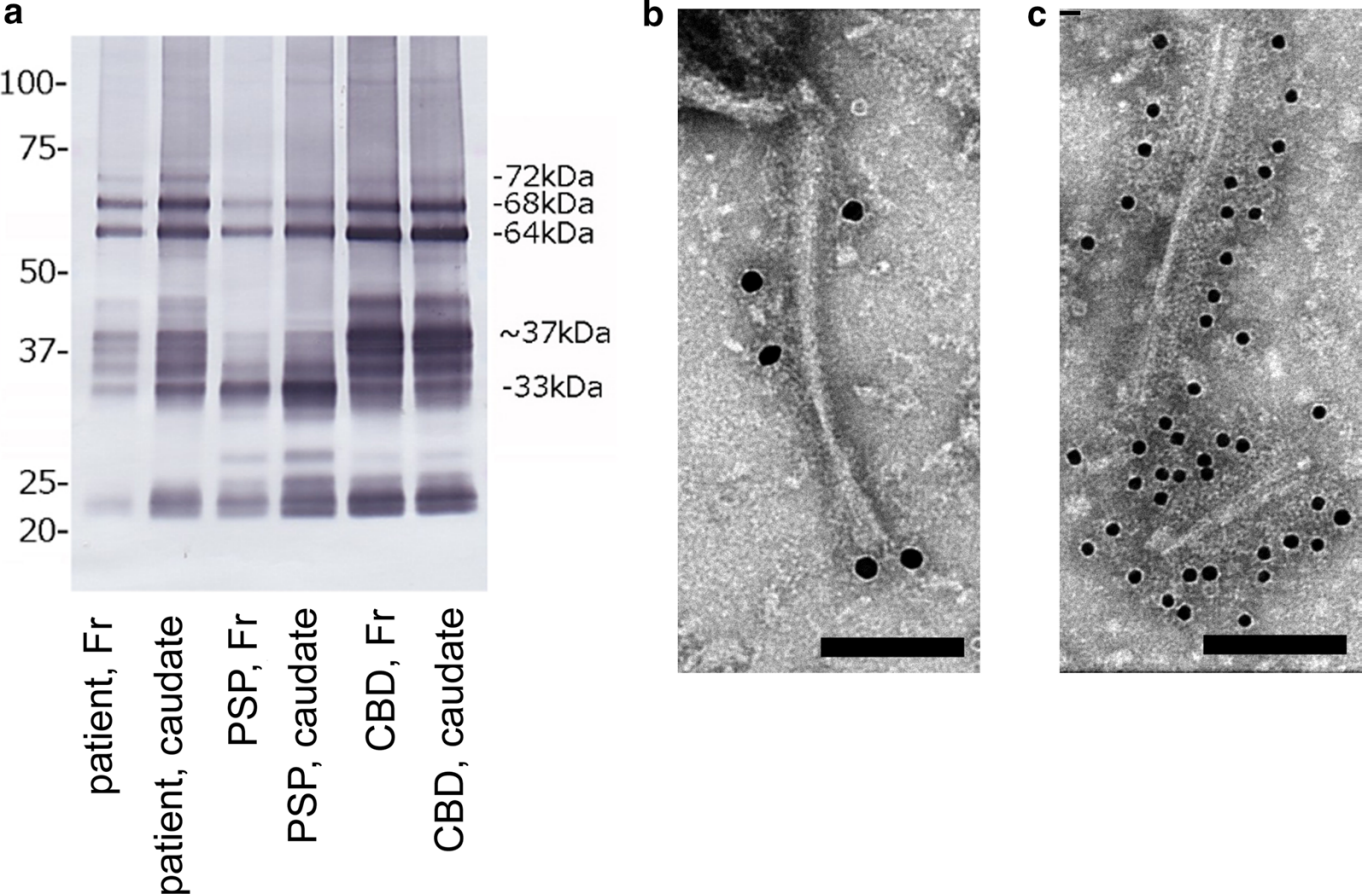
Western blot and immune electron microscopy of aggregated tau protein. **a** Sarkosyl-insoluble fractions of brain tissue lysates from the frontal cortex (Fr) and caudate nucleus of our patient were subjected to western blot analysis. Patients with progressive supranuclear palsy (PSP) and corticobasal degeneration (CBD) were also assessed for comparison. Western blot analysis using anti-T46 antibody exhibited bands prominent at 64 kDa, 68 kDa, 33 kDa, and 37 kDa. The bands at 33 kDa and 37 kDa were equally intense in our patient, whereas those at 33 kDa or 37 kDa were prominent in the patient with PSP or CBD, respectively. **b**, **c** The sarkosyl-insoluble fraction contained ribbon-like filaments with long periodicity, which were immunopositive for AT8 (**b**) and an anti-Tau C antibodies (**c**). Scale bars = 100 nm, original magnification × 60,000

Anti-tau (T46) immunoblot of sarkosyl-insoluble fractions from the frontal cortex and striatum revealed major doublets at 64 and 68 kDa and bands at 33 and 37 kDa. The blotting bands at 33 kDa and 37 kDa were equally prominent. These blotting patterns were different from those of PSP or CBD, which would be characterized by a prominent band at either 33 kDa or 37 kDa, respectively [2] (Fig. 5a).

### Outcomes of immunoelectron microscopy

Tau fibrils, which were isolated from the sarkosyl-insoluble fraction, had diameters of 15–20 μm and exhibited long periodicity and immunoreactivity to anti-AT8 and anti-tau C terminal antibodies (Fig. 5b, c).

### Outcomes of genetic analysis

There were no known pathogenic or rare variants in the causative genes related to Perry syndrome, FTD, familial Parkinson’s disease, parkinsonism, or Alzheimer’s disease.

## Discussion and conclusions

The patient died of respiratory failure 14 months after the presentation of parkinsonism. A postmortem study revealed neuronal and glial aggregation of 4R-tau. Tau aggregations and neuronal loss were prominent in the medial temporal lobes, the temporal tip, the striatum, the substantia nigra, the tegmentum of the midbrain, and the medullary reticular formation. Astrocytic inclusions comprised granular or dense aggregations of the proximal or distal portion of the foot processes; the morphological features were similar to those of the bush-like or thorn-shaped astrocytes in ARTAG [19]. Oligodendroglia showed the appearance of coiled bodies but not globular glial inclusions [17]. Western blot analysis of the sarkosyl-insoluble fraction revealed a low molecular band equally prominent at 33 and 37 kDa [2]. On electron microscopy, the aggregated tau fibrils were relatively straight with long periodicity, which was similar to those in PSP and CBD [3]. These clinical findings, morphological and biochemical findings of tau aggregations, and distributions of tau aggregations and neuronal loss could not be classified into known 4R-tau-related neurodegenerative disorders, including PSP, CBD, GGT, ARTAG, and AGD.

Her sibling also showed rapidly progressive parkinsonism and sudden death. Unfortunately, a postmortem study was not undertaken for him, and it was unclear if he also had 4R-tau aggregation. However, the familial clustering of neurological manifestations suggests a genetic background. We did not identify pathologic mutations in *MAPT* or *DCTN*-*1*, which are causative of familial tauopathy and Perry syndrome, respectively [6, 12]. The other genetic variants related to parkinsonism and cognitive disorders were also not detected either. We could not evaluate the possibility of a novel gene by whole genome sequencing or linkage analysis due to the small number of family members. We expect further investigation to find any novel gene variants, which characterize this unclassifiable 4R-tauopathy with clinically aggressive manifestations and the chance of familial history.

Sporadic or familial patients with unclassifiable 4R-tau aggregations have occasionally been reported [4, 7, 15, 18]. Although gene variants in *MAPT* or *FUS* have been suggested, the pathogenesis remains unclear [7, 18]. FTD, parkinsonism or both have been observed in previous reports. Important clinical features of our patient were nonmotor symptoms of progressive respiratory failure with hypercapnia, weight loss, and sleep disturbance. The postmortem assay revealed prominent aggregations of tau in the medullary reticular formation, particularly in the nucleus ambiguus. The region is known as a part of the ventral respiratory group and as a parasympathetic center. Its pathologic involvement might contribute to those nonmotor symptoms, as presumed for the autonomic dysfunctions of Perry syndrome [26]. Another finding of interest was severe tau aggregations in the lower motor neurons of the spinal cord. A subset of neuronal inclusions showed AT-8-immunopositive fibrous configurations that differed from pretangles or globose-type tangles. The skeletal muscles did not show neurogenic atrophy, and the lower motor neurons were not depleted; it remains unclear whether the tau aggregations of the lower motor neurons impaired muscle strength and ventilation. Prominent tau aggregation within the lower motor neurons has also been reported among patients with GGT, and those patients sometimes demonstrate motor neuron disease-like muscle weakness [8]. The limitation of this study is lack of comprehensive neurophysiological assessments including electromyography, respiratory monitoring, and polysomnography. Detailed assessments of the respiratory and autonomic systems could further reveal the pathomechanism of the various nonmotor symptoms of the patient.

Argyrophilic grains may contribute to atrophy and the presence of ballooned neurons in the medial temporal structures of our patient. Several studies have demonstrated patients with occasional diffuse AGD (DAGD) that involves not only the medial temporal lobes but also the neocortices, limbic cortices, and the brainstem [14, 22]. FTD-like behavioral disorders, mood disorders, or mild parkinsonism have been described in these patients [14, 22]. A recent study revealed that extensions of granular or fuzzy astrocytes were positively correlated with progression of AGD [23]. However, neuronal loss and neuronal and astrocytic tau aggregations in our patient were clearly broader than the presence of argyrophilic grains. The western blot of our patient exhibited a low molecular band equally prominent at 33 and 37 kDa, whereas that of AGD patients would show quite weak bands in those molecular weights [23]. We consider that the clinicopathological phenotypes of our patient cannot be explained by AGD alone. A prominent tau aggregation in the brainstem or the spinal cord could be indicative of IgLON5-related tauopathy. However, this disease entity usually displays a combination of 3R and 4R-tauopathy [9]. Myotonic dystrophy is also associated with tauopathies but usually demonstrates 3R tauopathy or a combination of 3R and 4R tauopathies [5, 20, 25, 32]. In addition, skeletal muscle of our patient did not have pathological features of myotonic dystrophy. Those facts differentiate our patient from IgLON5-related or myotonic dystrophy-related tauopathy, although assessments of HLA haplotypes or genetic analyses of DMPK and CNBP genes were not available. Neuronal aggregations of phosphorylated TDP-43 were influent but always colocalized with tau aggregations in our patient. The results of anti-p62 and anti-dipeptide immunohistochemistry and a C9orf72 gene analysis suggest that TDP-43 aggregation in our patient might be induced by 4R-tau aggregation [16] rather than primary TDP-43 proteinopathy.

In this report, we described a patient who was characterized by parkinsonism and progressive respiratory failure in association with unclassified 4R-tau aggregation. Our results further extend the clinical and neuropathologic spectra of 4R-tauopathy.

## Data Availability

All clinical data and material from the pathology are available from Mari Yoshida (myoshida@aichi-med-u.ac.jp).

## Abbreviations

4R-tau: four-repeat tau
3R-tau: three-repeat tau
AGD: argyrophilic grain dementia
ARTAG: aging-related tau astrogliopathy
CA: cornu ammonis
CBD: corticobasal degeneration
ChAT: choline acetyltransferase
DCTN-1: dynactin subunit 1
DNA: deoxyribonucleic acid
FTD: frontotemporal dementia
GGT: globular glial tauopathy
MAPT: microtubule-associated protein tau
PiD: Pick’s disease
PSEN-1: presenilin-1
PSP: progressive supranuclear palsy
TDP-43: TAR DNA binding protein 43 kDa
TH: tyrosine hydroxylase
TrOH: tryptophan hydroxylase
VLM nucleus: ventrolateral medullary nucleus
